# Supplementary material for: Dual heterogeneous interfaces enhance X-ray excited persistent luminescence for low-dose 3D imaging
Source: Nat Commun. 2024 Feb 7;15:1140. doi: 10.1038/s41467-024-45390-0 (PMC10850100; doi:10.1038/s41467-024-45390-0)
Supplement: Supplementary file 1 — Supplementary Information [file 41467_2024_45390_MOESM1_ESM.pdf]

Supplementary Information

Dual heterogeneous interfaces enhance X-ray excited persistent luminescence for low-dose 3D imaging

Lei Lei[1], Minghao Yi[1], Yubin Wang[1], Youjie Hua[1], Junjie Zhang[1], Paras N. Prasad[2], Shiqing Xu[1]

[1]Key Laboratory of Rare Earth Optoelectronic Materials and Devices of Zhejiang Province, Institute of Optoelectronic Materials and Devices, China Jiliang University, Hangzhou 310018, People's Republic of China

[2]Institute for Lasers, Photonics, and Biophotonics and Department of Chemistry, University at Buffalo, State University of New York, Buffalo, New York 14260, United States

Correspondence and requests for materials should be addressed to Lei Lei (email: leilei@cjlu.edu.cn) or to P.N.Prasad (email: pnprasad@buffalo.edu) or to Shiqing Xu (email: shiqingxu@cjlu.edu.cn)

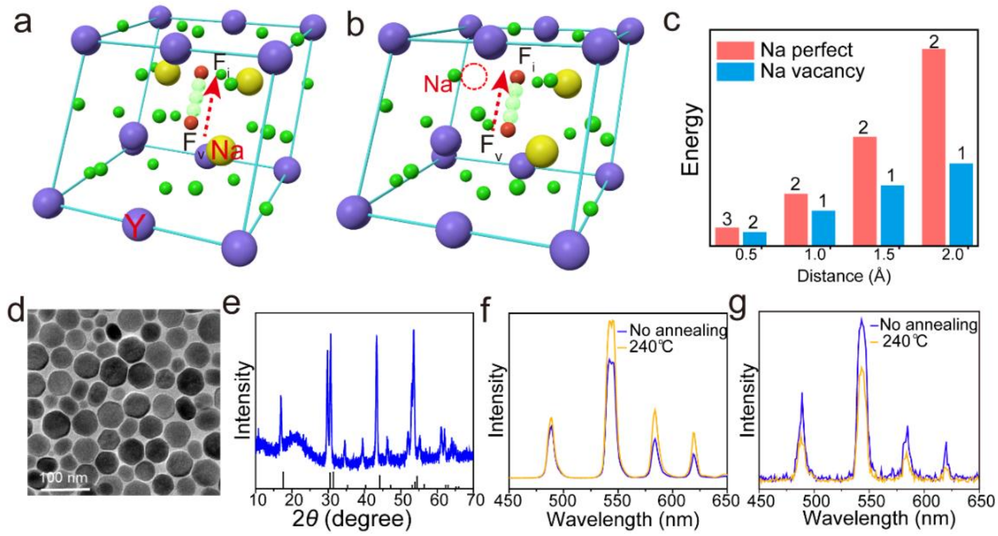

Supplementary Fig. 1 (a) Crystal structure of perfect $NaLuF_4$. (**b**) Crystal structure of $NaLuF_4$ with Na^+ vacancies. (**c**) Calculated Frenkel defect formation energies in these two crystal structures. TEM image (**d**) and XRD pattern (**e**) of the as-prepared $NaLuF_4$:15Tb NPs. The bars represent the standard data of hexagonal $NaLuF_4$ (JCPDS No. 270726). X-ray excited optical luminescence (**f**) and XEPL (**g**) intensity variations of the $NaLuF_4$:15Tb NPs before and after annealing. Source data are provided as a Source Data file.

Supplementary Note 1. Density functional theory. DFT was employed to calculate the formation energies (E_f) of anion Frenkel defects in $NaLuF_4$ crystals, both with and without Na^+ vacancies. When dislocating F^- ions into interstitial sites at various separation distances (0.5, 1.0, 1.5, and 2.0 Å), the E_f values were consistently smaller for the F^- ions with fewer coordination numbers. Consequently, F^- ions with incomplete coordination on the surface are more inclined to form Frenkel defects compared to their fully coordinated counterparts.

Furthermore, it is noteworthy that the X-ray excited optical luminescence intensity of $NaLuF_4$:15Tb NPs increased following wet chemical annealing[1], while its XEPL intensity decreased. This enhancement can be attributed to the rearrangement of surface ions and the subsequent reduction in surface defects. Considering the XEPL mechanism, the stronger XEPL observed in case of incomplete surface ion arrangement can be attributed to an increase in F^- related Frenkel defects and, consequently, a greater number of trapped electrons. These analyses provide further evidence that incompletely coordinated surface F^- ions indeed facilitate the formation of Frenkel defects upon exposure to ionizing radiation.

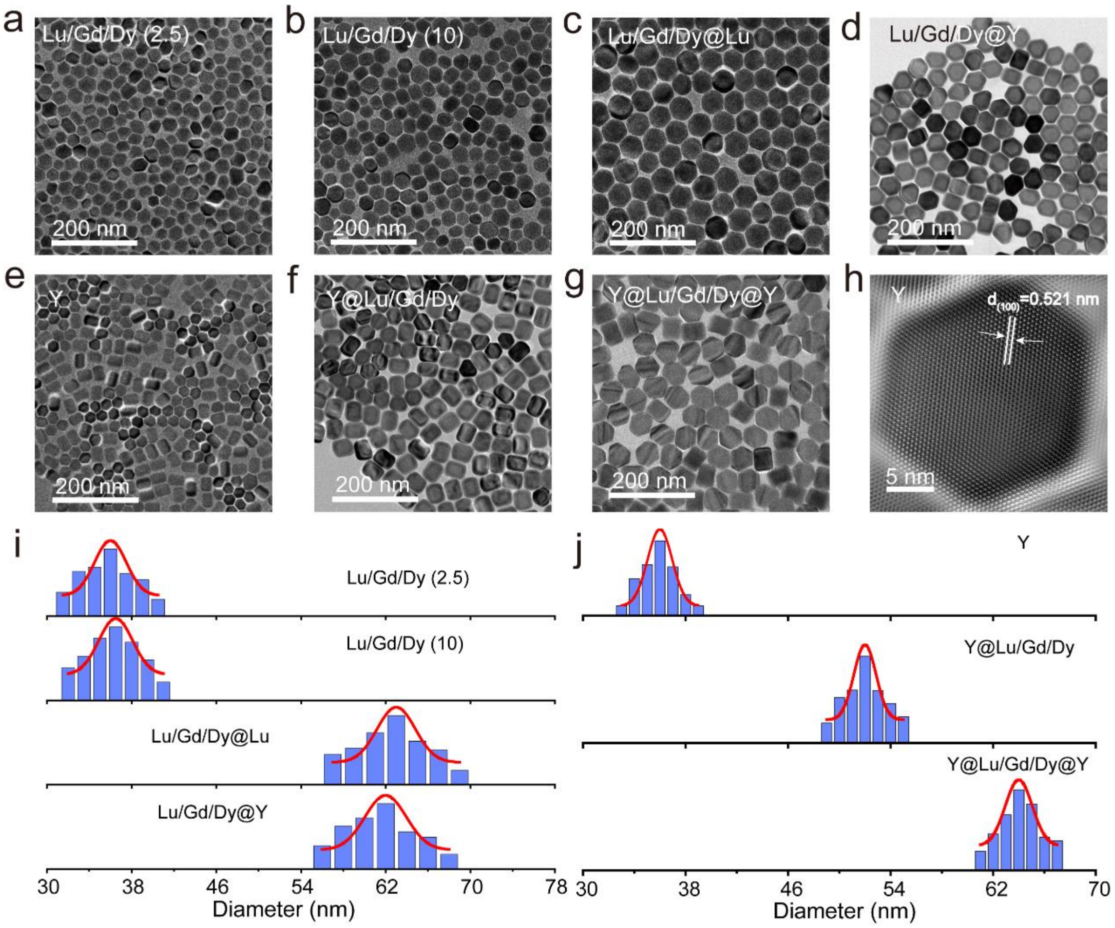

Supplementary Fig. 2 TEM images of the Lu/Gd/Dy ([Na]/[RE] = 2.5) (**a**), Lu/Gd/Dy ([Na]/[RE] = 10) (**b**), Lu/Gd/Dy@Lu (**c**), Lu/Gd/Dy@Y (**d**), $NaYF_4$ (**e**), Y@Lu/Gd/Dy (**f**), Y@Lu/Gd/Dy@Y (**g**) NPs. **h** High resolution TEM image of an individual $NaYF_4$ NP. **i-j** Histograms of size distributions corresponding to the NPs shown in (a-g). 80-100 NPs are counted. Source data are provided as a Source Data file.

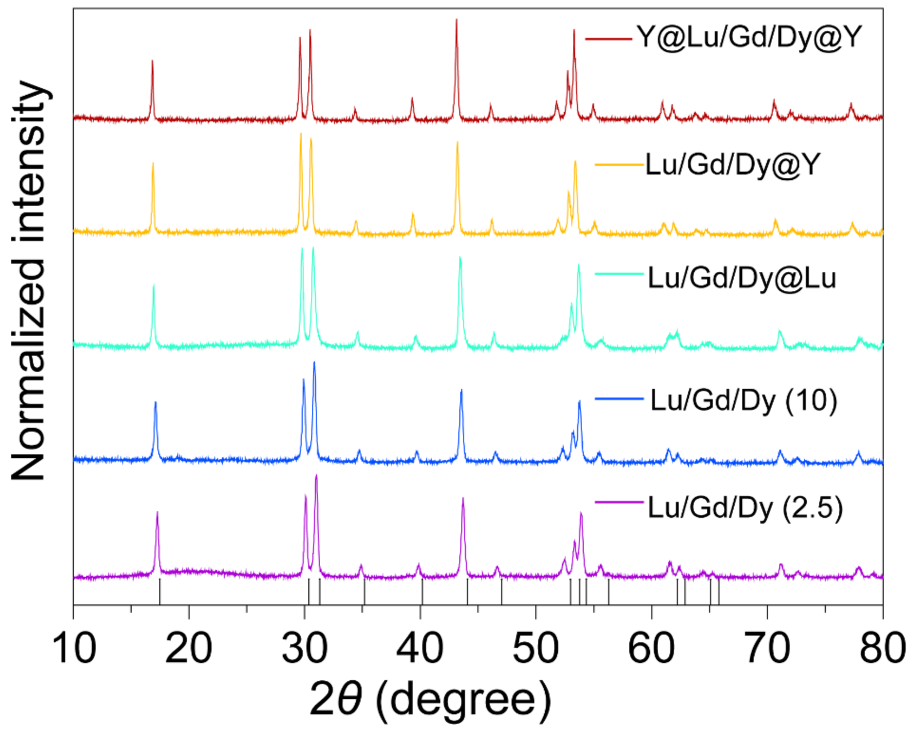

Supplementary Fig. 3 XRD patterns of the Lu/Gd/Dy ([Na]/[RE] = 2.5), Lu/Gd/Dy ([Na]/[RE] = 10), Lu/Gd/Dy@Lu, Lu/Gd/Dy@Y, Y@Lu/Gd/Dy@Y NPs. The bars represent the standard data of hexagonal $NaLuF_4$ (JCPDS No. 270726). Source data are provided as a Source Data file.

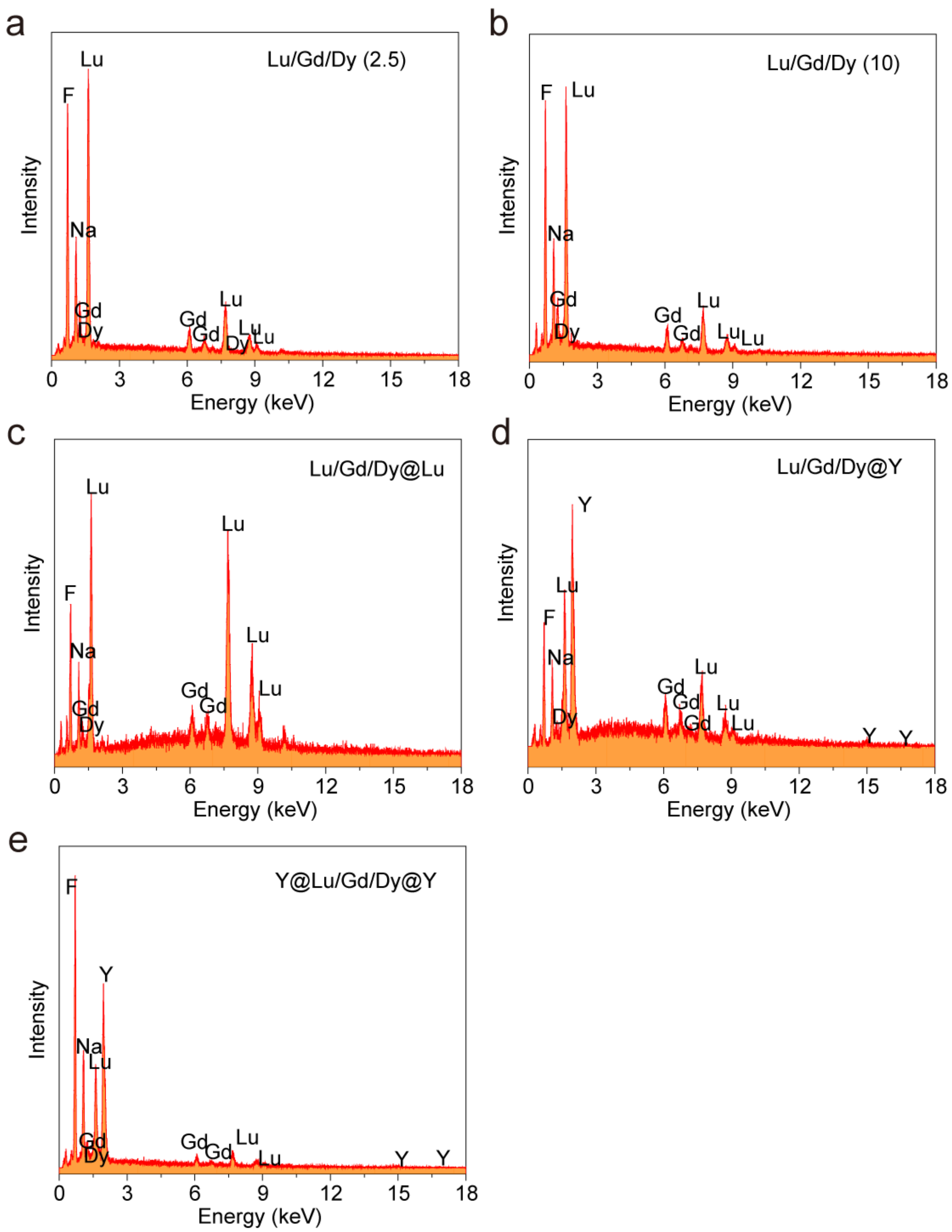

Supplementary Fig. 4 EDX spectra of the Lu/Gd/Dy ([Na]/[RE] = 2.5) (**a**), Lu/Gd/Dy ([Na]/[RE] = 10) (**b**), Lu/Gd/Dy@Lu (**c**), Lu/Gd/Dy@Y (**d**), Y@Lu/Gd/Dy@Y (**e**) NPs.

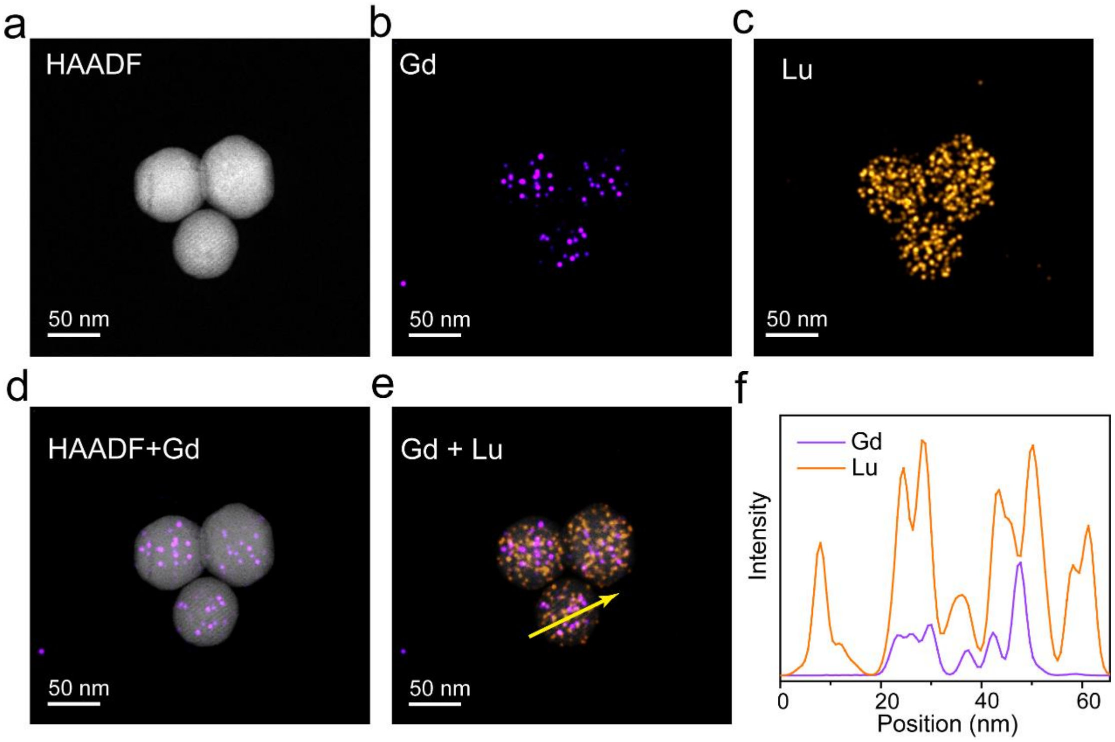

Supplementary Fig. 5 HAADF image (**a**), element mapping results, Gd (**b**, purple) and Lu (**c**, yellow), mixed HAADF image and Gd signal (**d**), mixed Gd and Lu signals (**e**), EDX line scan (**f**), of the Lu/Gd/Dy@Lu core@shell NPs. The line scan is along the direction indicated by the yellow arrow. Source data are provided as a Source Data file.

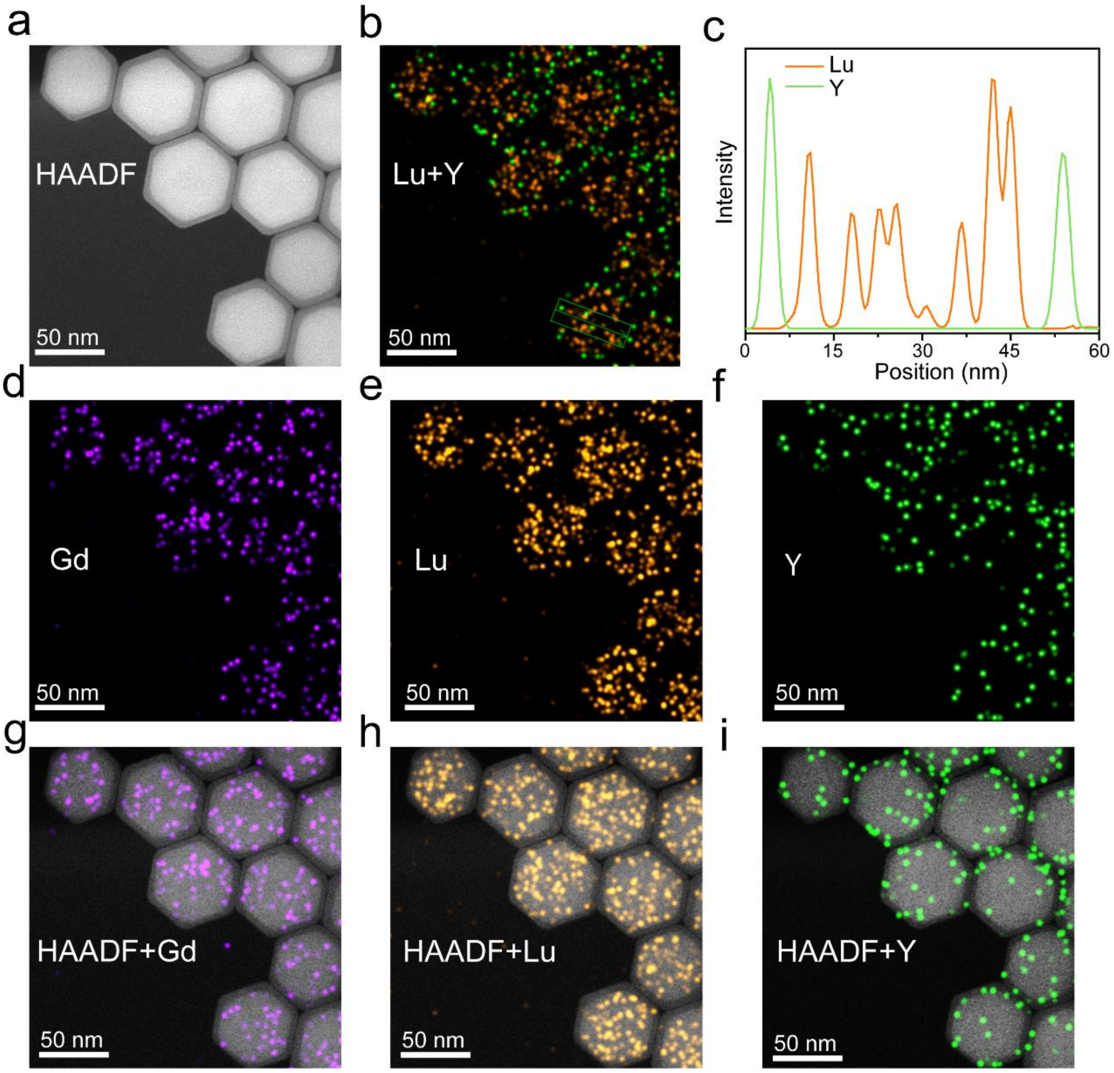

Supplementary Fig. 6 HAADF image (**a**), mixed Lu (yellow) and Y (green) signals (**b**), EDX line scan (**c**), element mapping results (**d, e, f**), mixed HAADF image and Gd (purple) signal (**g**), mixed HAADF image and Lu signal (**h**), mixed HAADF image and Y signal (**i**), of the Lu/Gd/Dy@Y core@shell NPs. Source data are provided as a Source Data file.

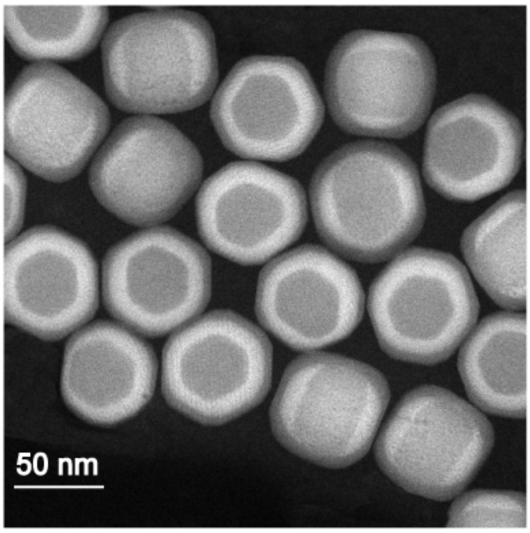

Supplementary Fig. 7 HAADF image of the Y@Lu/Gd/Dy@Y NPs corresponding to the image presented in Fig. 1b.

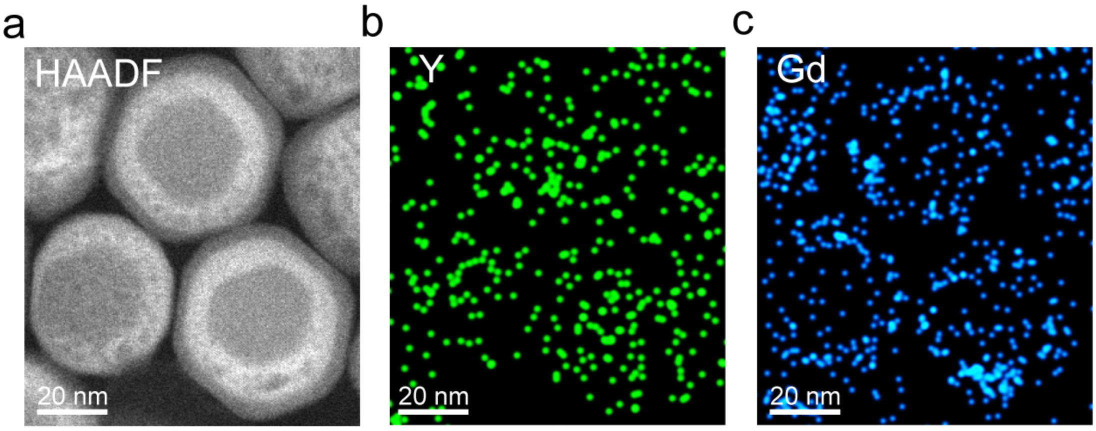

Supplementary Fig. 8 HAADF image (a), and the corresponding element mapping results of the Y@Lu/Gd/Dy@Y NPs. Green, Y signal; blue, Gd signal.

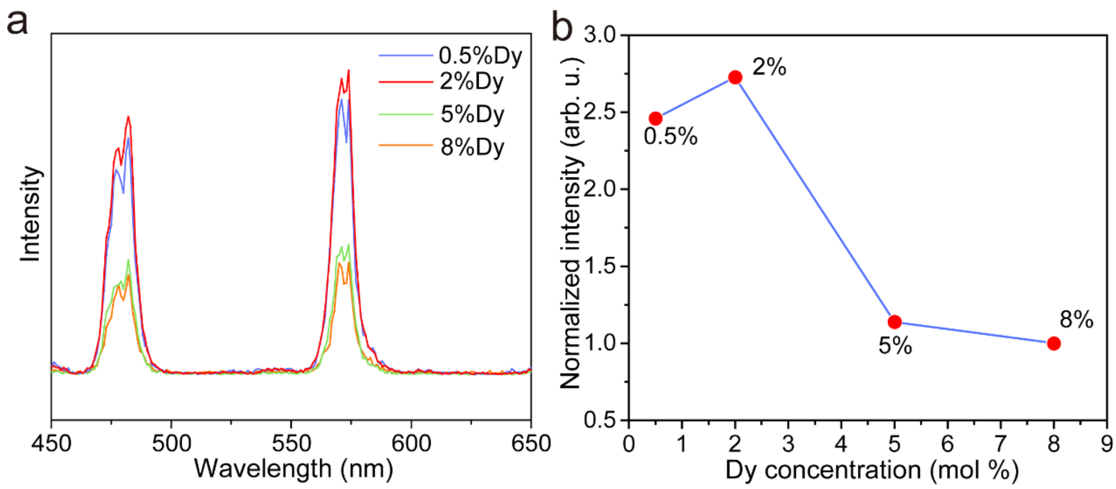

Supplementary Fig. 9 XEPL spectra (**a**) and the corresponding normalized integral XEPL intensities (**b**) of the Y@Lu/Gd/Dy@Y NPs with different Dy doping concentrations (0.5, 2, 5, 8 mol%). Source data are provided as a Source Data file.

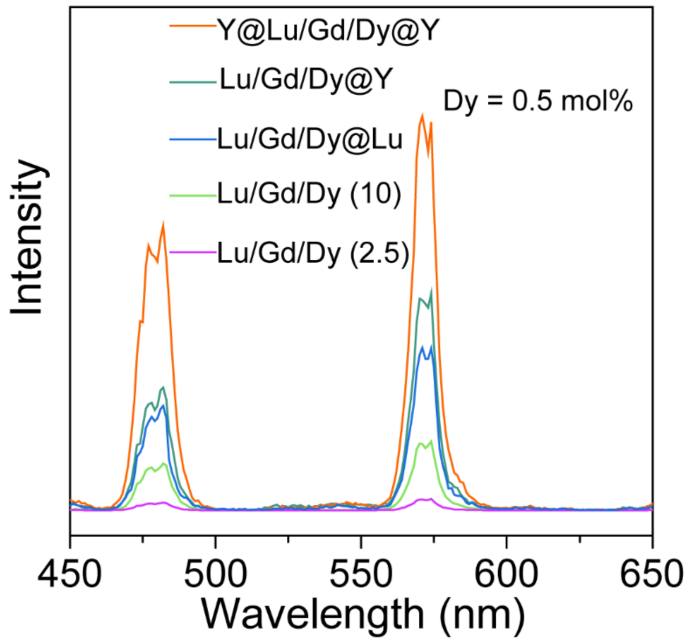

Supplementary Fig. 10 XEPL spectra of the Lu/Gd/Dy ([Na]/[RE] = 2.5), Lu/Gd/Dy ([Na]/[RE] = 10), Lu/Gd/Dy@Lu, Lu/Gd/Dy@Y, Y@Lu/Gd/Dy@Y NPs. The Dy doping concentration in these NPs were 0.5 mol%. Source data are provided as a Source Data file.

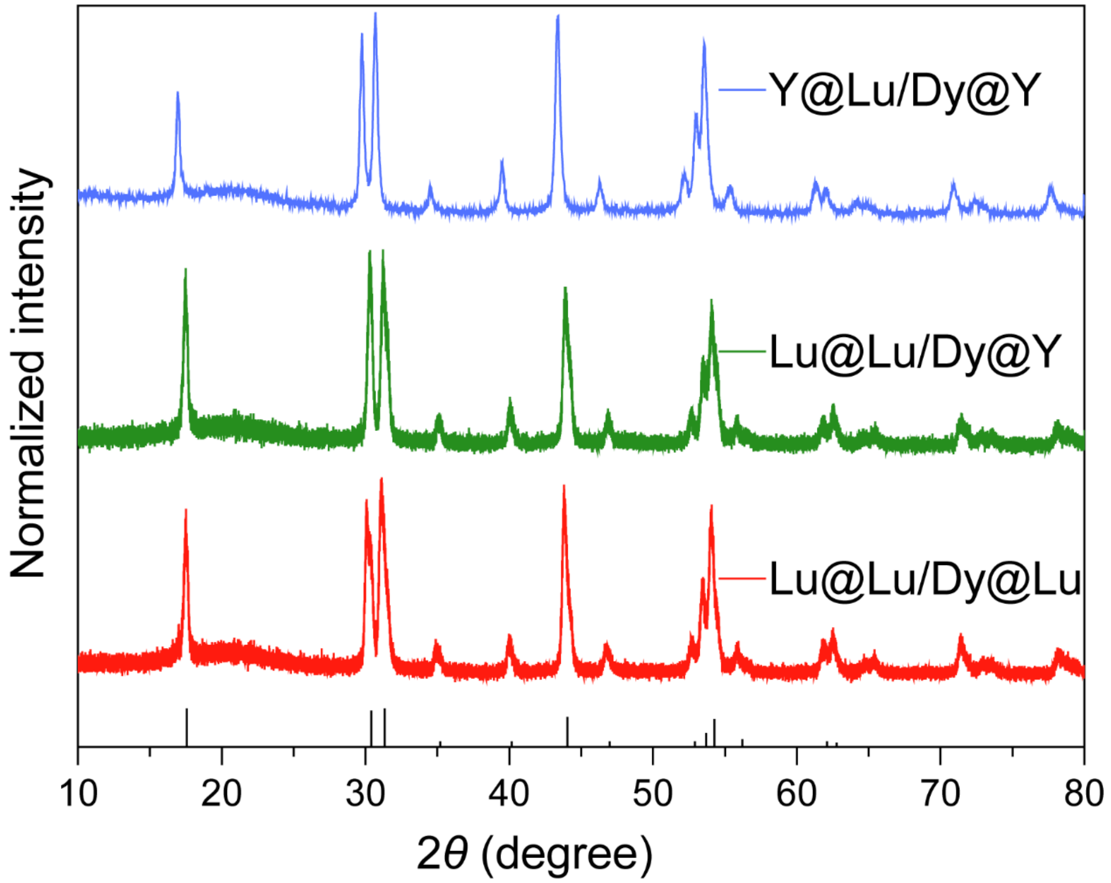

Supplementary Fig. 11 XRD patterns of the Lu@Lu/Dy@Lu, Lu@Lu/Dy@Y and Y@Lu/Dy@Y core@shell@shell NPs. The bars represent the standard data of hexagonal $NaLuF_4$ (JCPDS No. 270726). Source data are provided as a Source Data file.

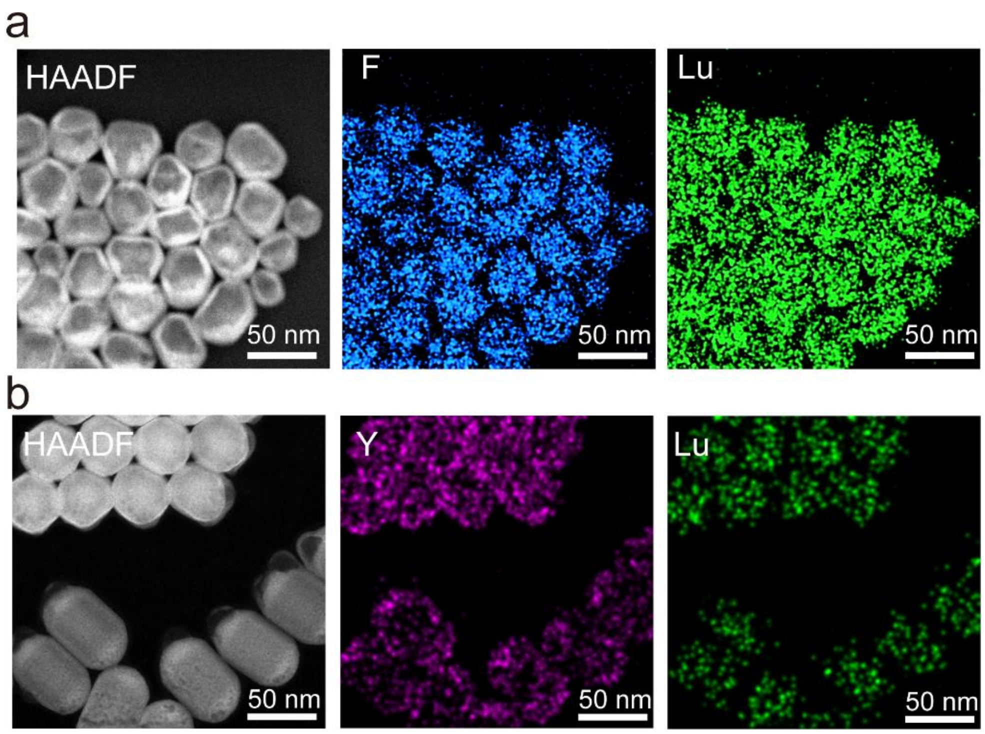

Supplementary Fig. 12 HAADF images and corresponding element mapping results of the Lu@Lu/Dy@Lu (**a**) and Lu@Lu/Dy@Y (**b**) core@shell@shell NPs. Blue, F signal; green, Lu signal; purple, Y signal.

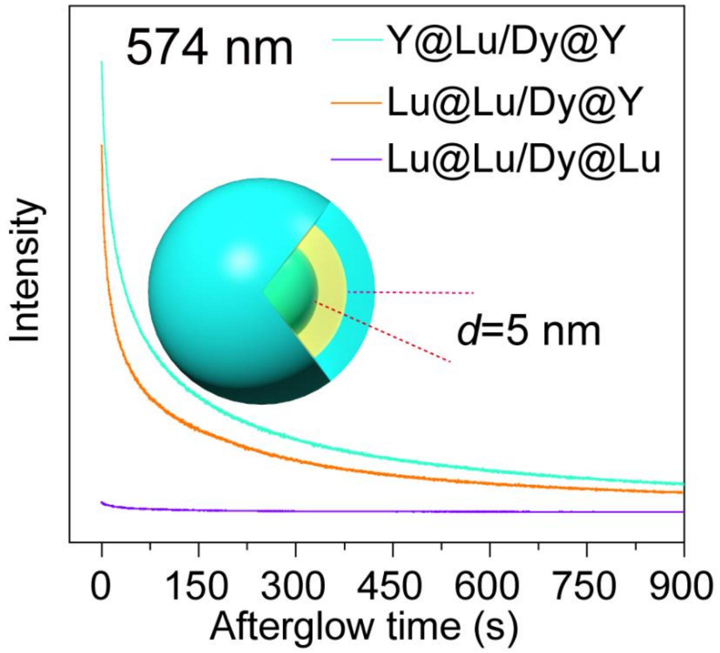

Supplementary Fig. 13 XEPL decay curves of the Lu@Lu/Dy@Lu, Lu@Lu/Dy@Y and Y@Lu/Dy@Y NPs. The interlayer thickness is about 5 nm. Source data are provided as a Source Data file.

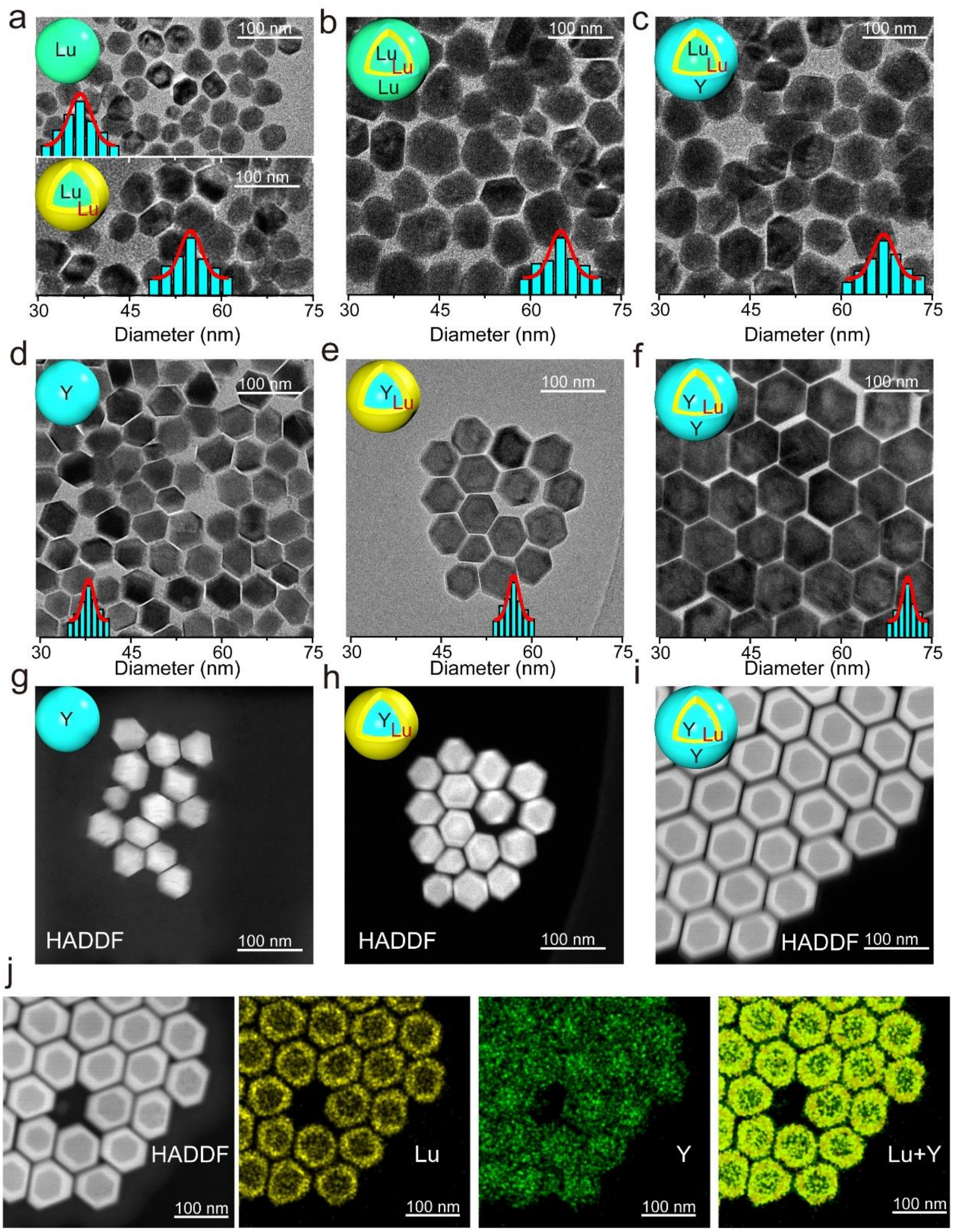

Supplementary Fig. 14 TEM images of the $NaLuF_4$ **(a, top)**, Lu@Lu/Dy **(a, bottom)**, Lu@Lu/Dy@Lu **(b)**, Lu@Lu/Dy@Y **(c)**, $NaYF_4$ **(d)**, Y@Lu/Dy **(e)**, Y@Lu/Dy@Y **(f)**. HAADF images of the $NaYF_4$ **(g)**, Y@Lu/Dy **(h)**, Y@Lu/Dy@Y **(i)**. Insets are histograms illustrating size distributions of their corresponding 15-50 NPs. **j** HAADF image and corresponding element mapping results of the Y@Lu/Dy@Y core@shell@shell NPs. The interlayer thickness is about 9 nm. Yellow, Lu signal; green, Y signal.

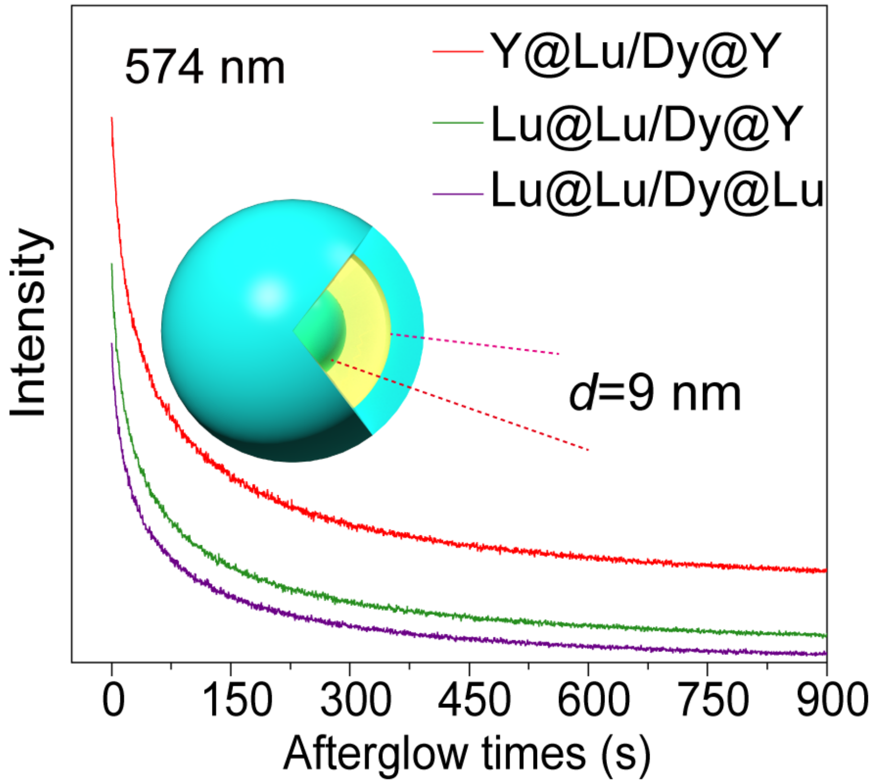

Supplementary Fig. 15 XEPL decay curves of the Lu@Lu/Dy@Lu (purple), Lu@Lu/Dy@Y (green) and Y@Lu/Dy@Y (red) NPs. The interlayer thickness is about 9 nm. Source data are provided as a Source Data file.

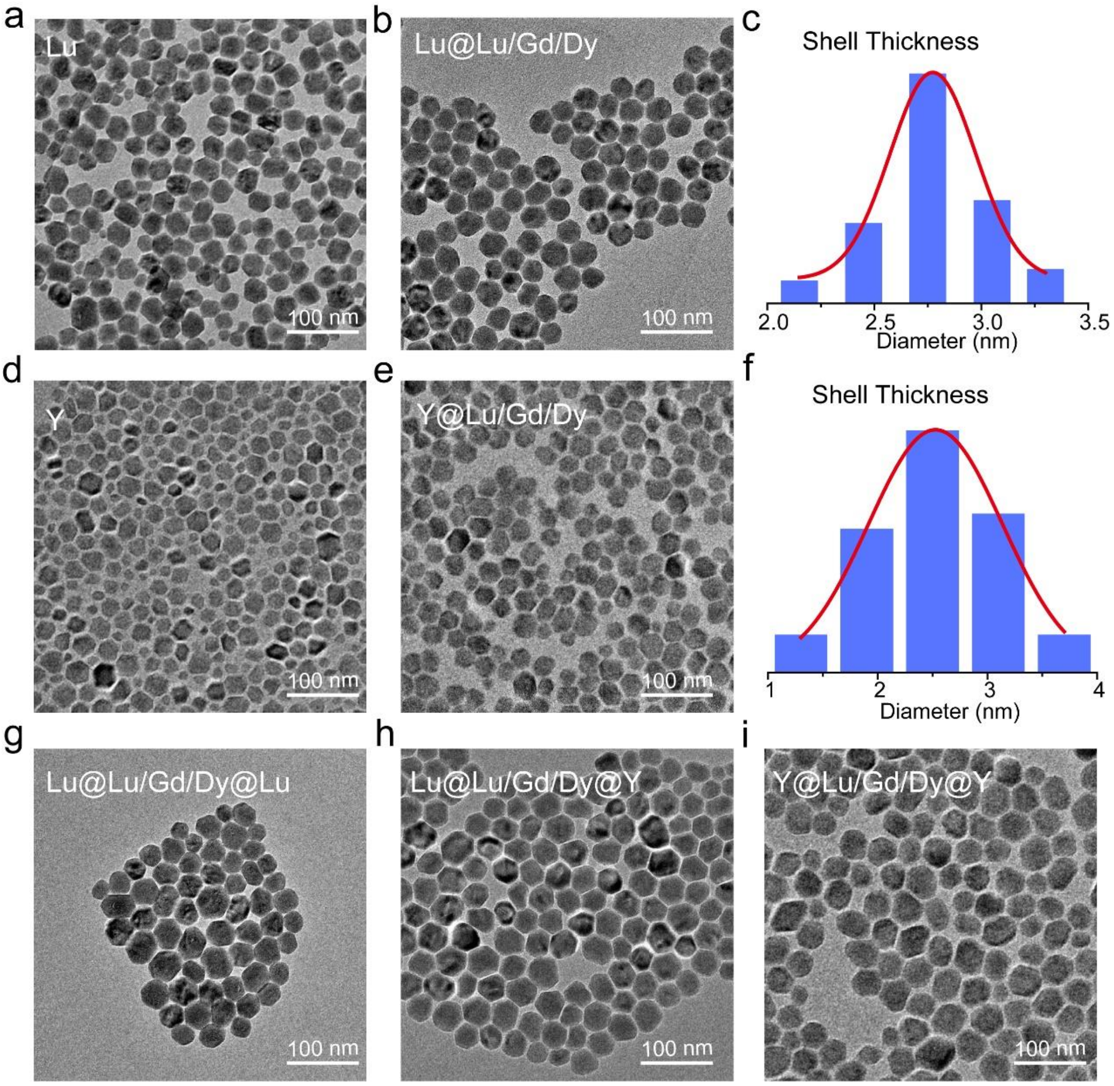

Supplementary Fig. 16 TEM images of the $NaLuF_4$ core (**a**), Lu@Lu/Gd/Dy core@shell NPs (**b**), and corresponding histogram of shell thickness distributions, 100 NPs are accounted (**c**). TEM images of the $NaYF_4$ core (**d**), Y@Lu/Gd/Dy core@shell NPs (**e**), and corresponding histogram of shell thickness distributions, 100 NPs are accounted (**f**). TEM images of the Lu@Lu/Gd/Dy@Lu (**g**), Lu@Lu/Gd/Dy@Y (**h**), Y@Lu/Gd/Dy@Y (**i**) core@shell@shell NPs.

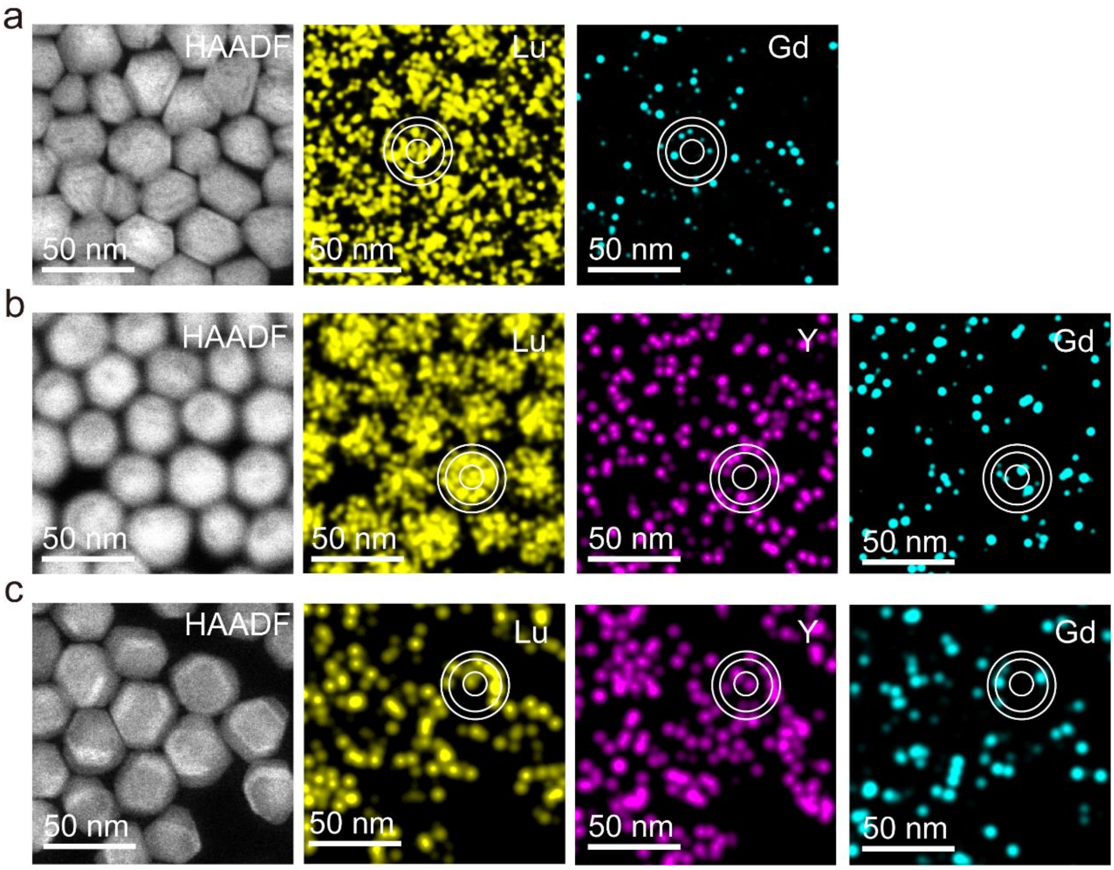

Supplementary Fig. 17 HAADF images and element mapping results of the Lu@Lu/Gd/Dy@Lu (**a**), Lu@Lu/Gd/Dy@Y (**b**) and Y@Lu/Gd/Dy@Y (**c**) core@shell@shell NPs. Yellow, Lu signal; cyan, Gd signal; purple, Y signal.

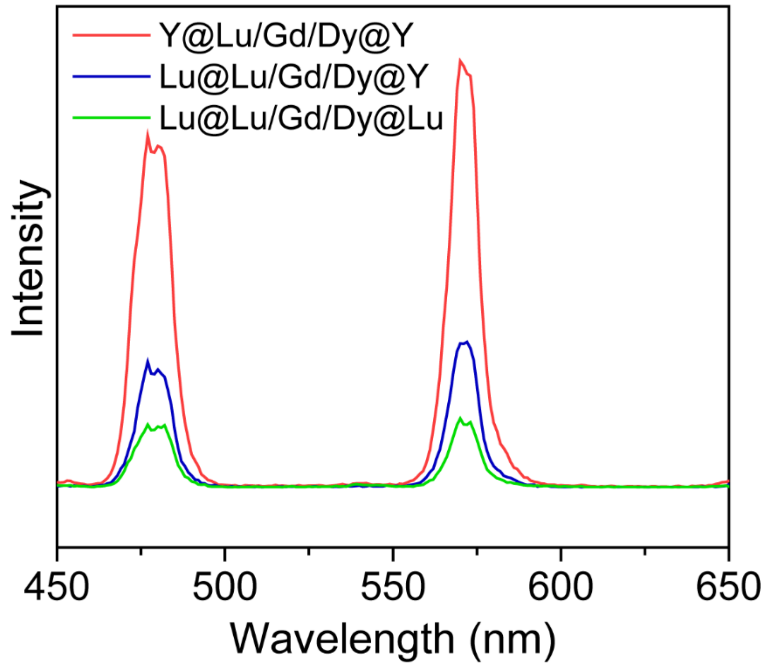

Supplementary Fig. 18 XEPL spectra of the Lu@Lu/Gd/Dy@Lu (green), Lu@Lu/Gd/Dy@Y (blue), Y@Lu/Gd/Dy@Y (red) core@shell@shell NPs. Source data are provided as a Source Data file.

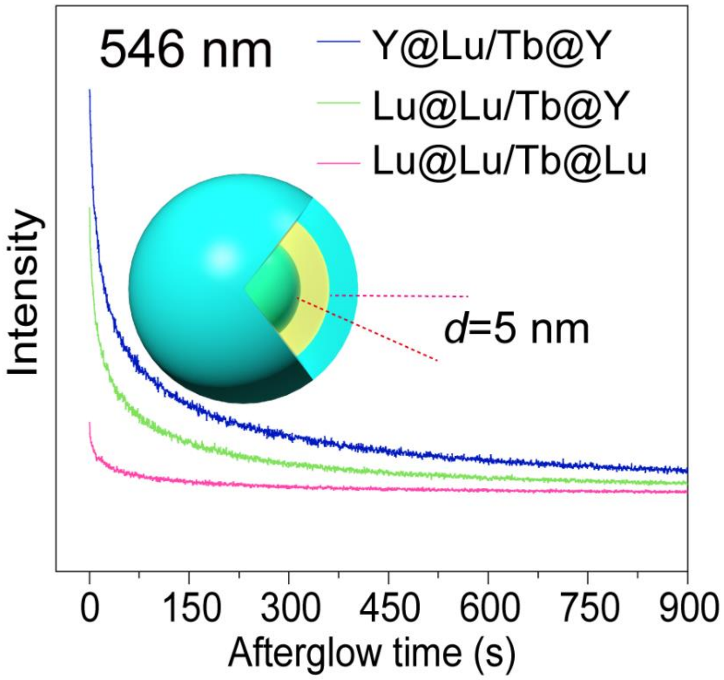

Supplementary Fig. 19 XEPL decay curves of the Y@Lu/Tb@Y (blue), Lu@Lu/Tb@Y (green) and Lu@Lu/Tb@Lu (pink) core@shell@shell NPs. Source data are provided as a Source Data file.

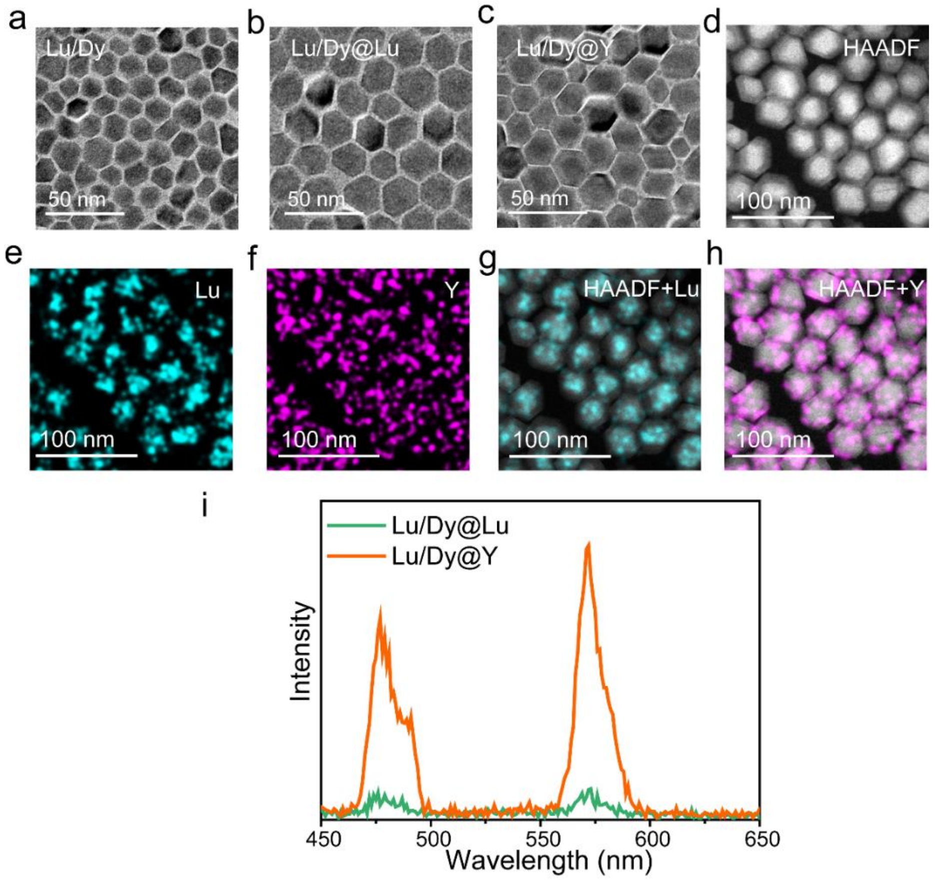

Supplementary Fig. 20 TEM images of the Lu/Dy core (**a**), Lu/Dy@Lu (**b**), Lu/Dy@Y (**c**) core@shell NPs. HAADF image (**d**), element mapping results (**e, f**), mixed HAADF image and Lu signal (**g**), mixed HAADF image and Y signal (**h**), of the Lu/Dy@Y core@shell NPs. Cyan, Lu signal; purple, Y signal. **i** XEPL spectra of the Lu/Dy@Lu (green) and Lu/Dy@Y (orange) core@shell NPs. Source data are provided as a Source Data file.

Supplementary Note 2. Cation intermixing. The cation intermixing issue during shell growth varies with the number density of core NPs [2]. We used core NPs with a low number density (2.09×10^{13} mL^{-1}) and a low Na^{+} concentration in the shell layer ([Na]/[RE] = 2.5) to prepare Lu/Dy@Lu and Lu/Dy@Y core@shell NPs. These two core@shell NPs share the same core size and exhibit similar shell thickness. It is evident that the XEPL intensity of heterogeneous Lu/Dy@Y core@shell NPs was substantially stronger than that of homogeneous Lu/Dy@Lu core@shell NPs. This observation underscores that the issue of cation intermixing does not alter the conclusion that heterogeneous interfaces contribute significantly to the enhancement of XEPL intensity.

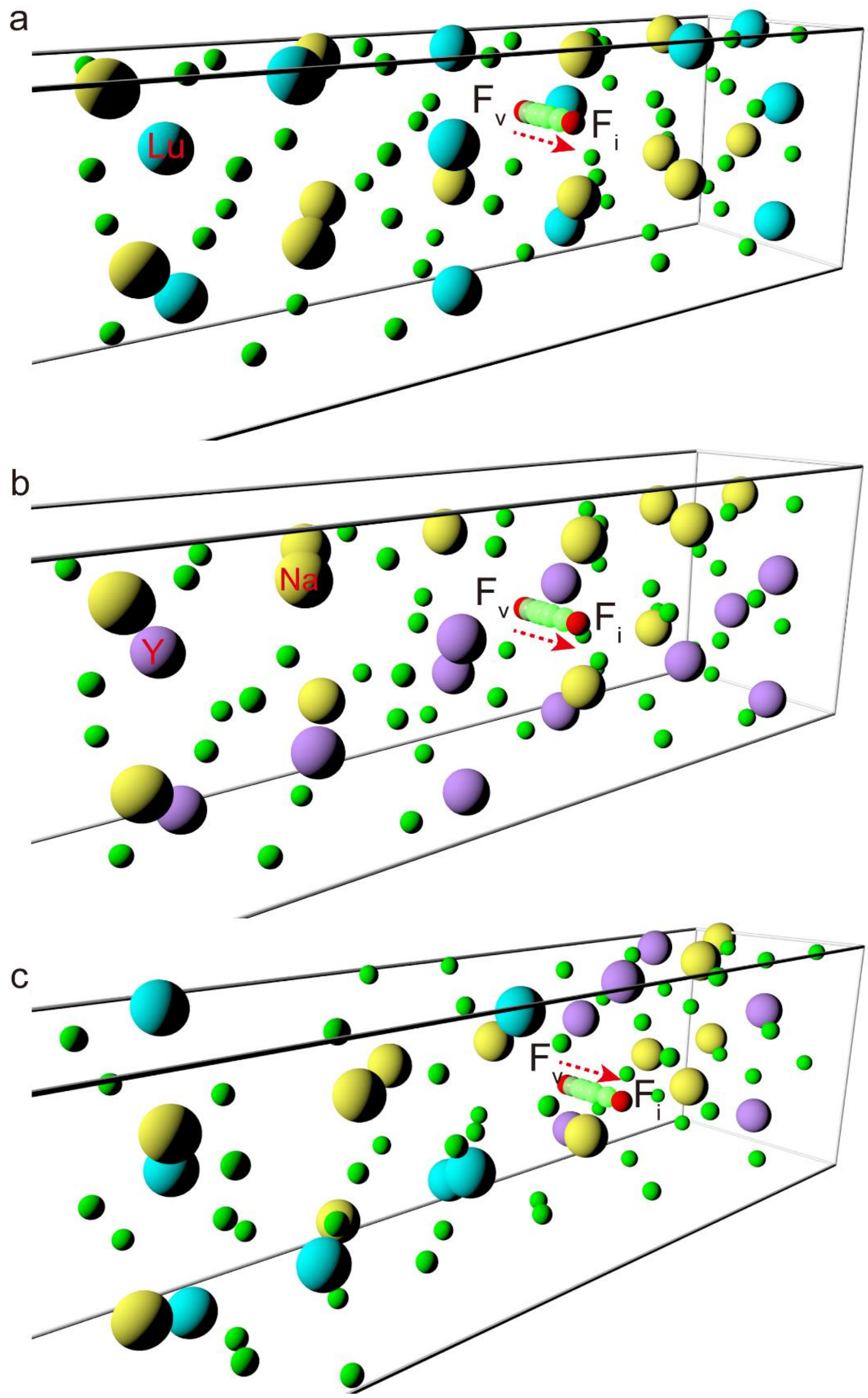

Supplementary Fig. 21 Crystal structures of the homogeneous Lu@Lu (**a**), Y@Y (**b**), and heterogeneous Lu@Y (**c**) systems. Yellow, Na atom; cyan, Lu atom; purple, Y atom; red, Frenkel defect.

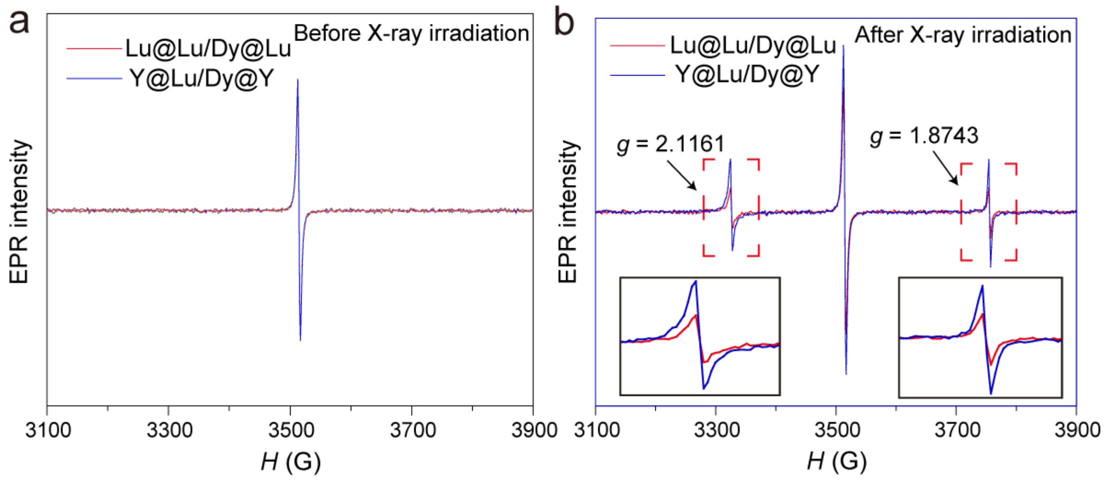

Supplementary Fig. 22 EPR spectra of the Lu@Lu/Dy@Lu (red line) and Y@Lu/Dy@Y (blue line) NPs, before X-ray irradiation (**a**) and after X-ray irradiation (**b**). *H*, magnetic field. Source data are provided as a Source Data file.

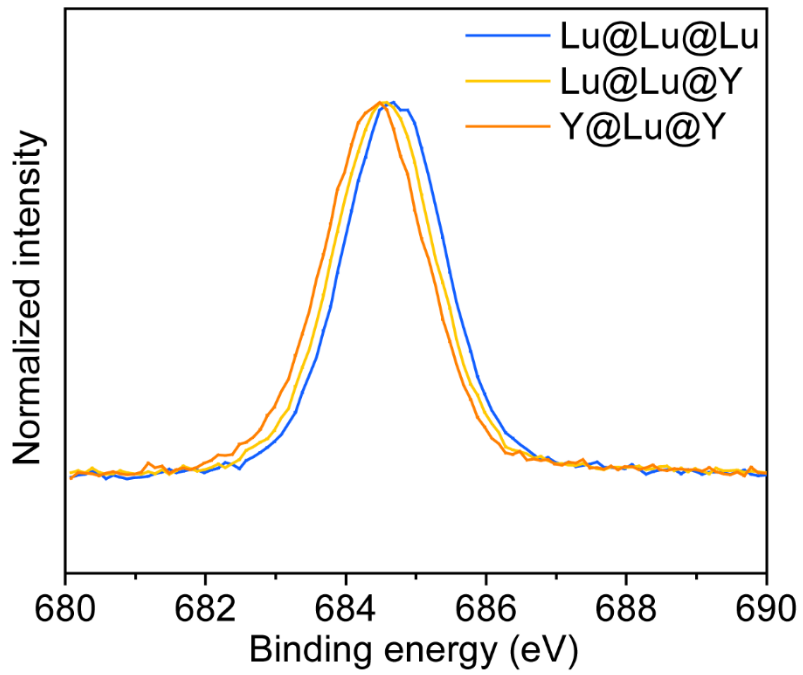

Supplementary Fig. 23 XPS spectra of F 1*s* in the Y@Dy/Lu@Y (orange), Lu@Lu/Dy@Y (yellow), and Lu@Lu/Dy@Lu (blue) core@shell@shell NPs. Source data are provided as a Source Data file.

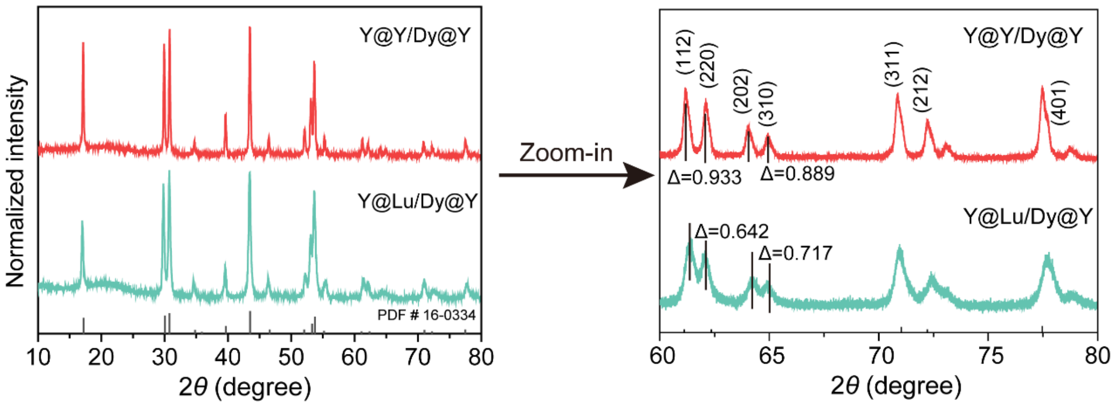

Supplementary Fig. 24 Slow-scan XRD patterns of the heterogeneous Y@Lu/Dy@Y and homogeneous Y@Y/Dy@Y core@shell@shell NPs. Step-size, 0.002. Source data are provided as a Source Data file.

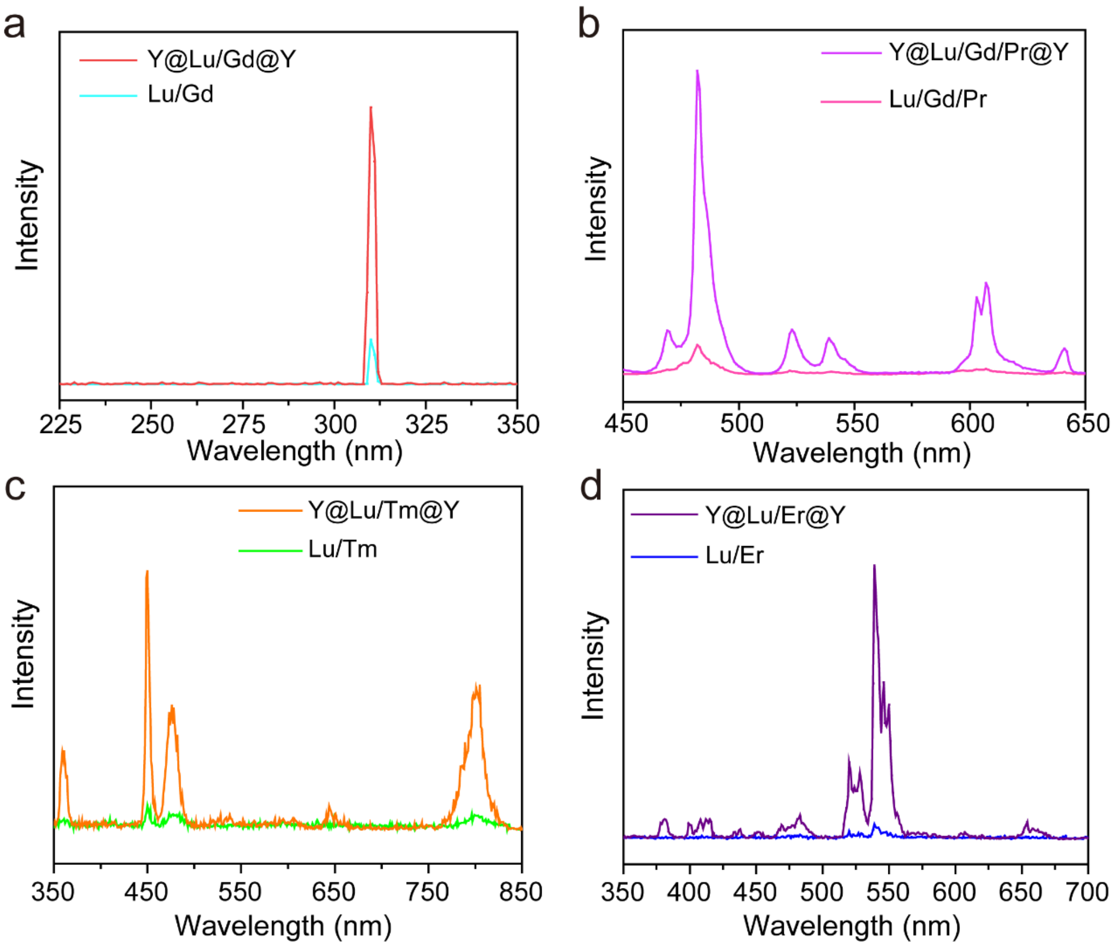

Supplementary Fig. 25 XEPL spectra of the Lu/Gd, Y@Lu/Gd@Y (**a**), Lu/Pr, Y@Lu/Pr@Y (**b**), Lu/Tm, Y@Lu/Tm@Y (c), Lu/Er, Y@Lu/Er@Y (**d**). The Gd, Pr, Tm, and Er doping concentrations were 20, 1, 1, 0.75 mol%, respectively. Source data are provided as a Source Data file.

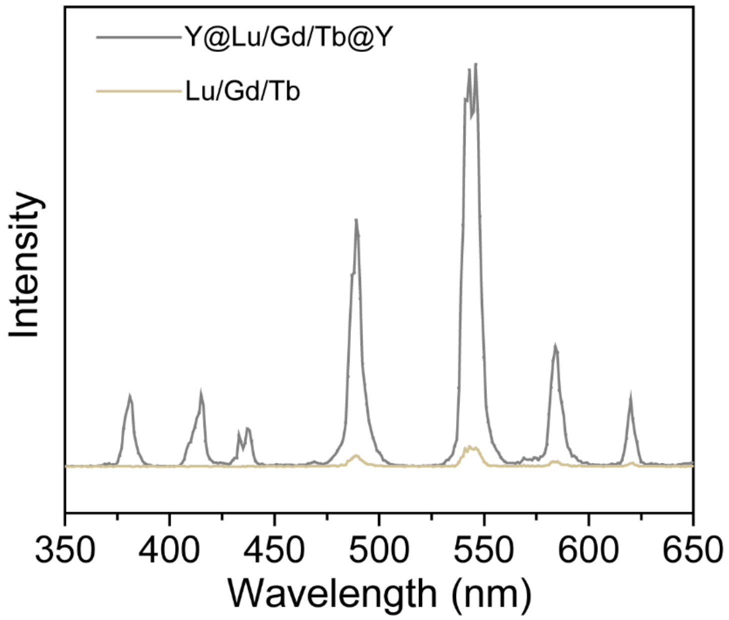

Supplementary Fig. 26 XEPL spectra of the Lu/Gd/Tb core and Y@Lu/Gd/Tb@Y core@shell@shell NPs. The Tb doping concentration was 15 mol%. Source data are provided as a Source Data file.

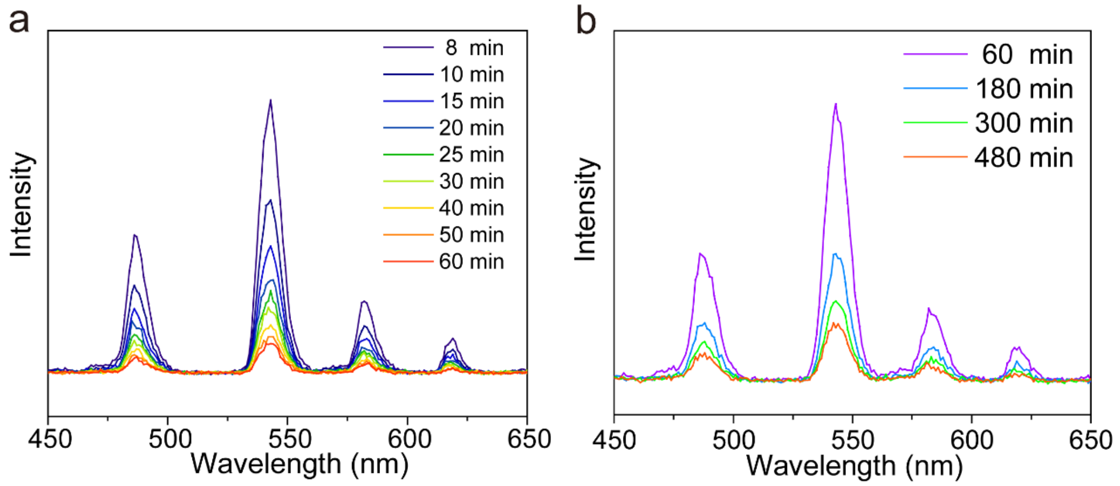

Supplementary Fig. 27 XEPL spectra of the Y@Lu/Gd/Tb@Y core@shell@shell NPs at different times after the cessation of X-rays. Source data are provided as a Source Data file.

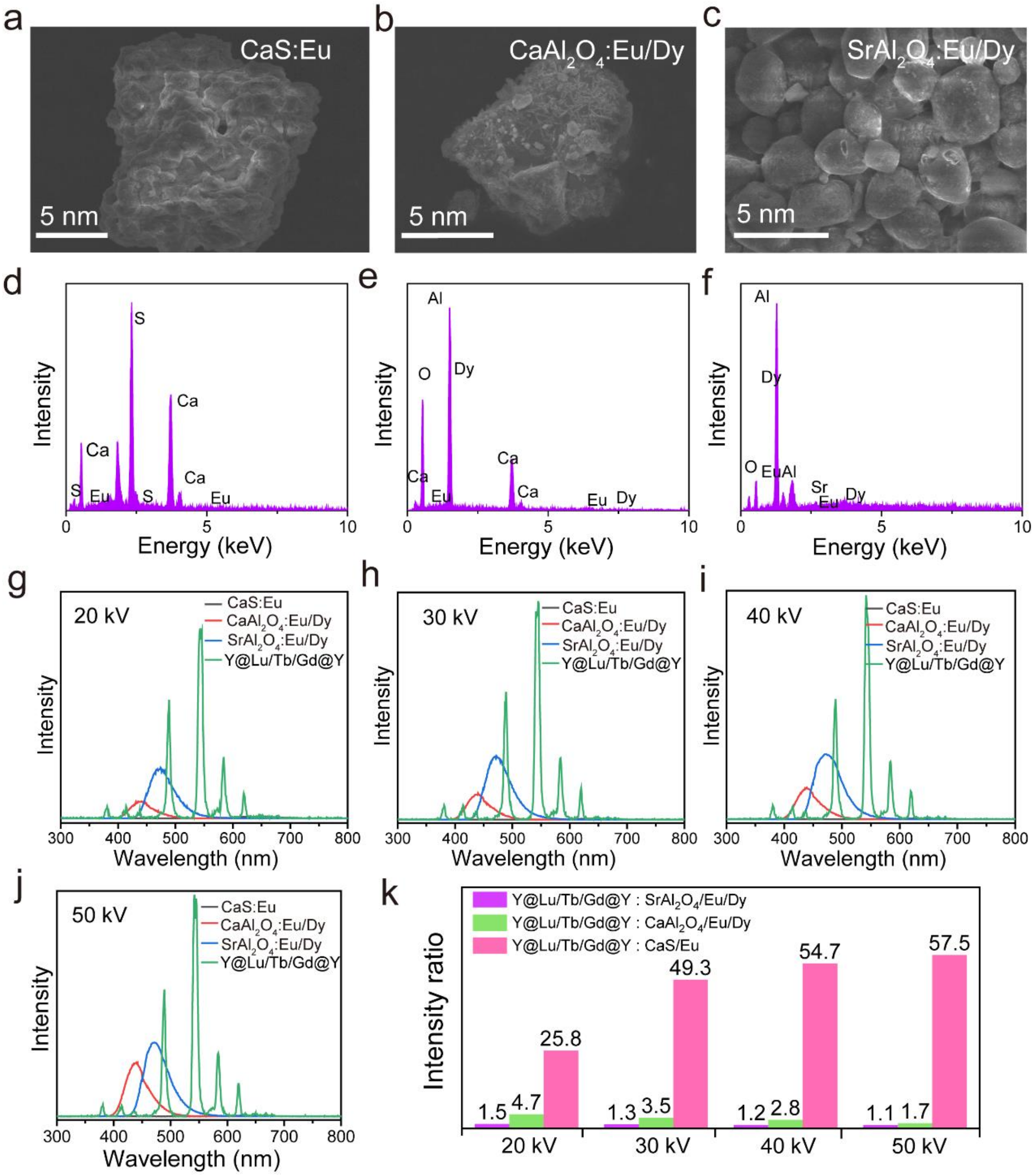

Supplementary Fig. 28 Scanning electron microscope images of commercial persistent phosphors, CaS:Eu (**a**), $CaAl_2O_4$:Eu/Dy (**b**), $SrAl_2O_4$:Eu/Dy (**c**). EDX spectra of the CaS:Eu (**d**), $CaAl_2O_4$:Eu/Dy (**e**), $SrAl_2O_4$:Eu/Dy (**f**). XEPL spectra under different irradiation conditions, 20 KV (**g**), 30 KV (**h**), 40 KV (**i**), 50 KV (**j**). **k** Compared XEPL intensity ratios under different irradiation conditions. Source data are provided as a Source Data file.

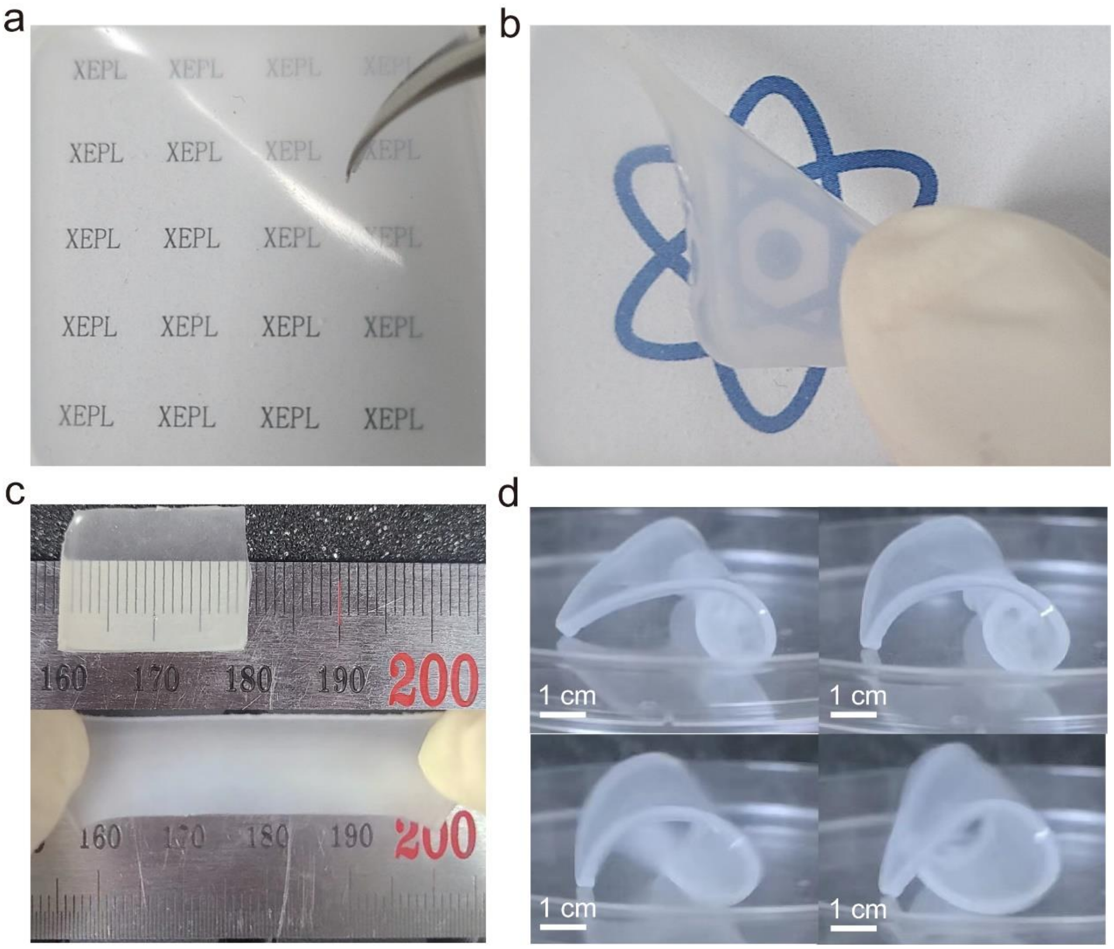

Supplementary Fig. 29 Photographs of the original (**a**), folded (**b**), stretched (**c**), and bent (**d**) Y@Lu/Gd/Tb@Y core@shell@shell NPs integrated flexible film.

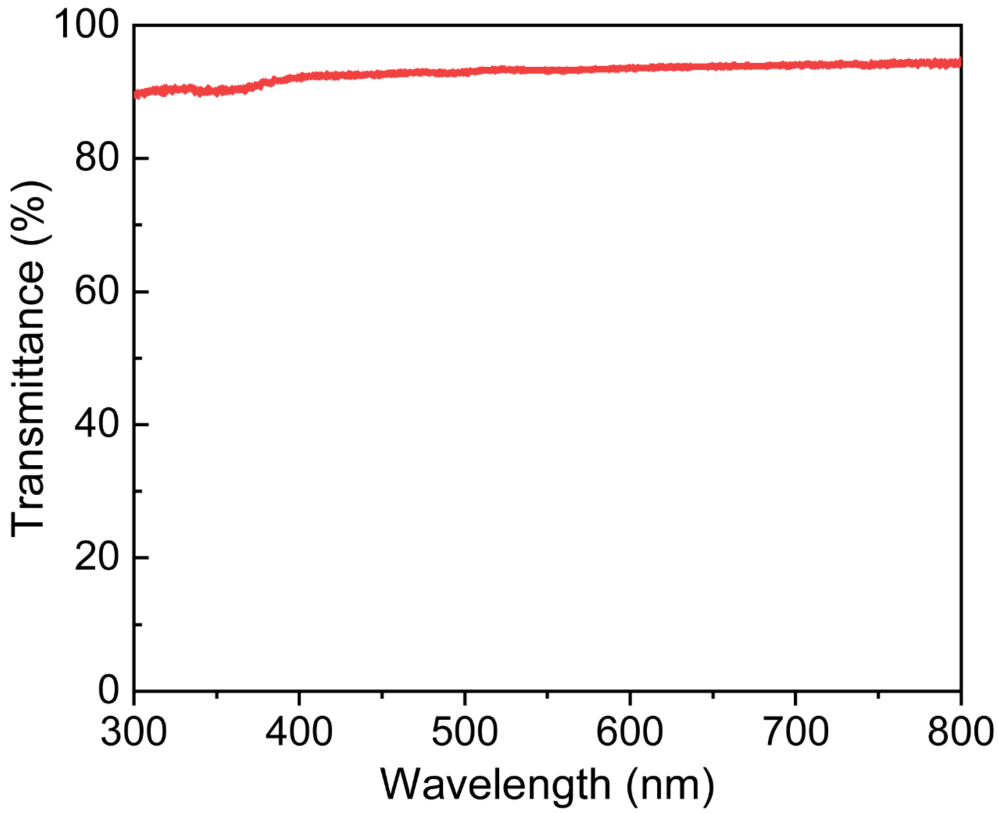

Supplementary Fig. 30 Transmittance spectrum of the flexible film. Source data are provided as a Source Data file.

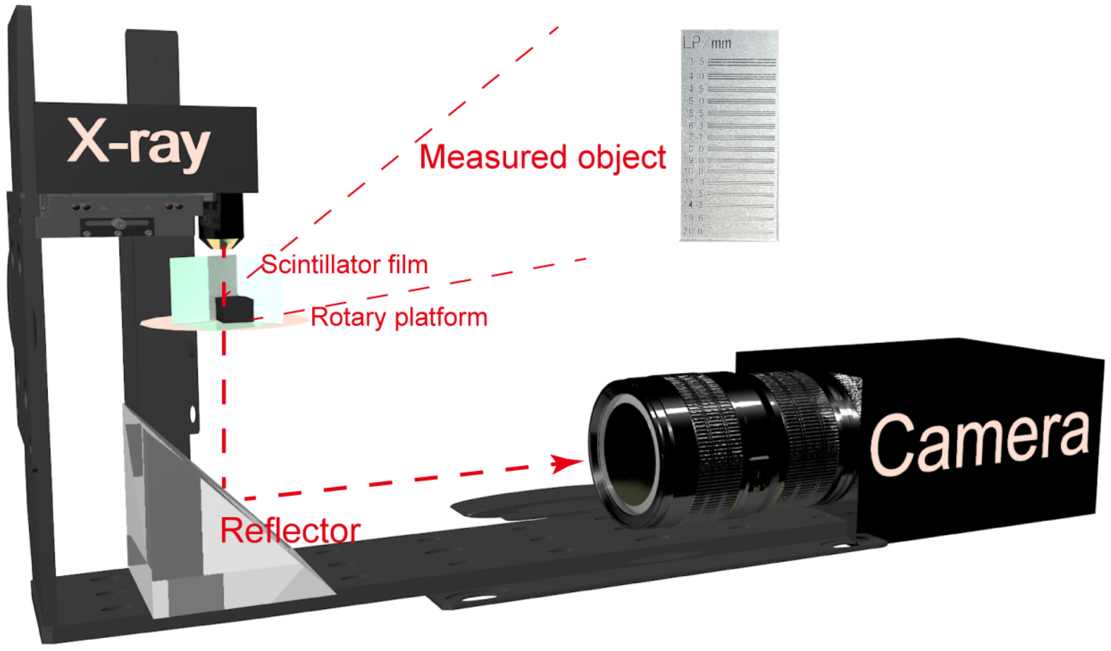

Supplementary Fig. 31 Schematic illustration of the X-ray imaging system.

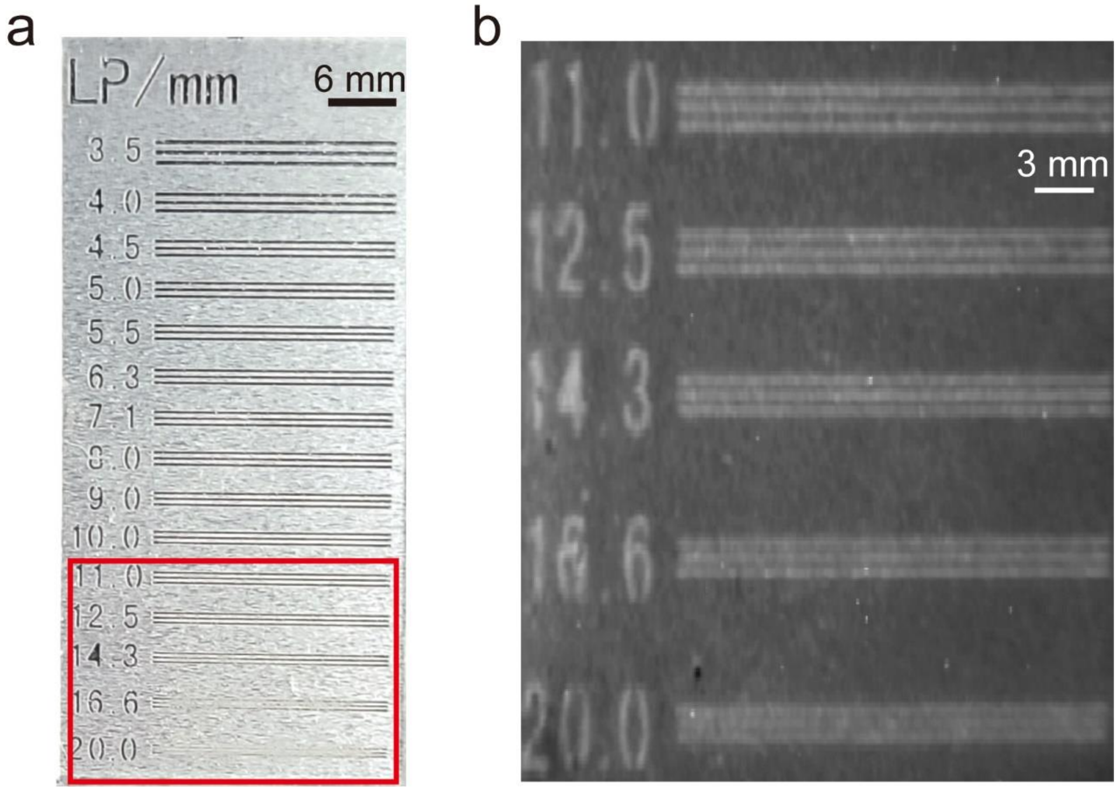

Supplementary Fig. 32 Photograph (**a**) and real-time X-ray image (**b**) of a standard X-ray resolution pattern plate.

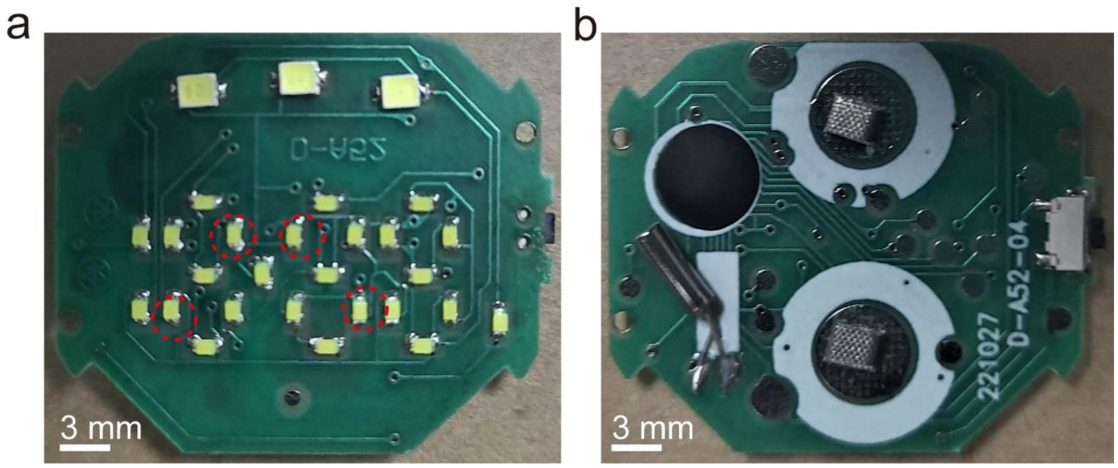

Supplementary Fig. 33 a-b Photographs of the utilized electronic watch.

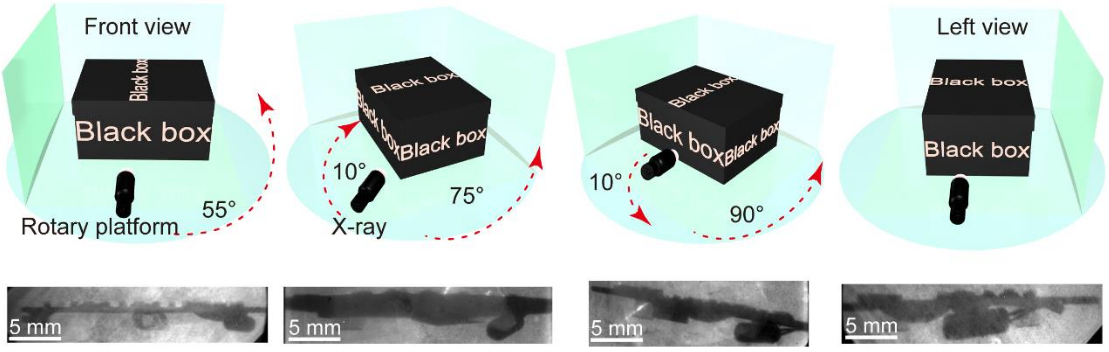

Supplementary Fig. 34 Delayed XEPL-based images from different directions.

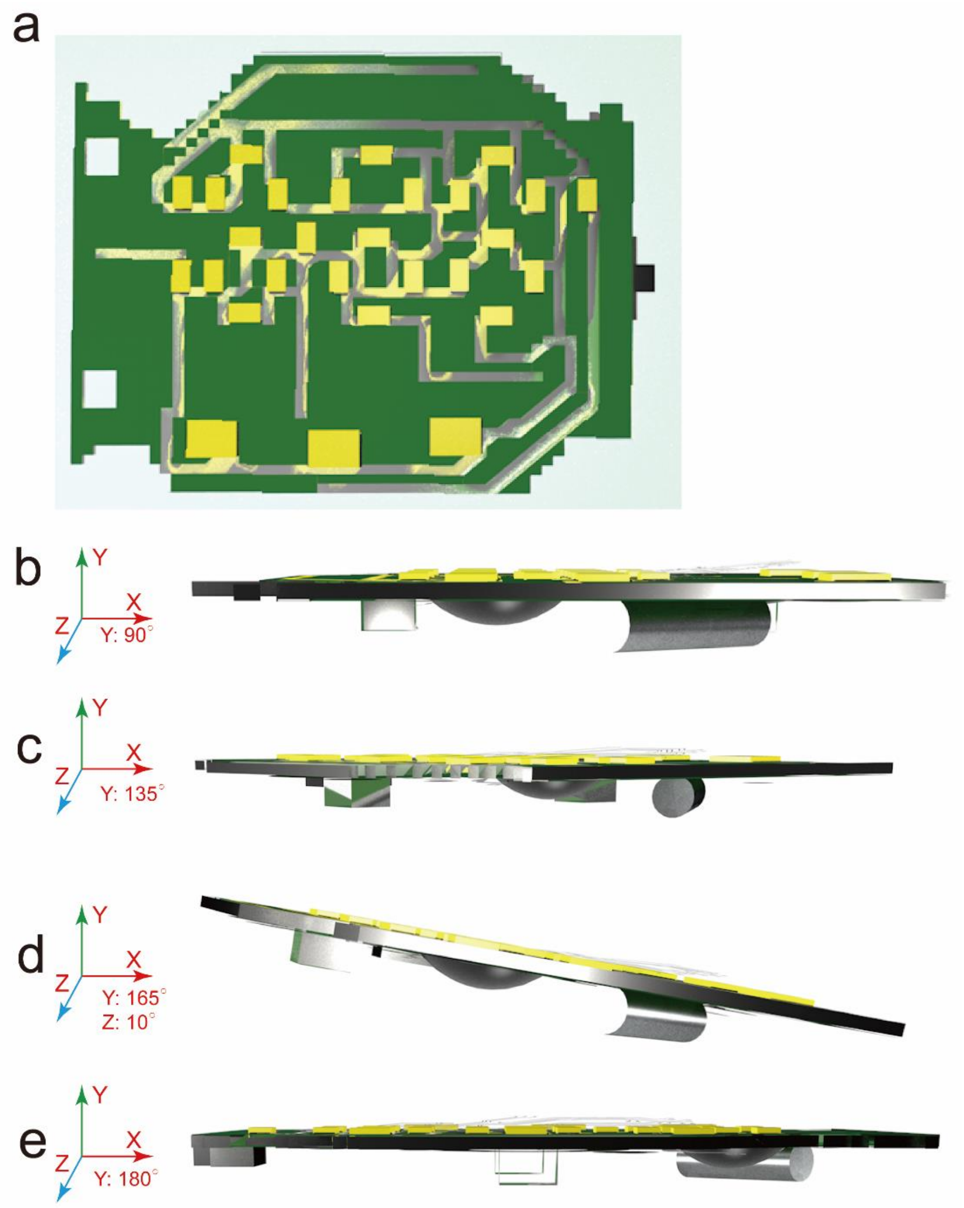

Supplementary Fig. 35 Reconstructed models based on the delayed images from different directions.

Supplementary Note 3. The parameters of the camera. Pixel size: 6.5 μm × 6.5 μm; Effective number of pixels: 2304 × 2304; Full frame: 2304 × 6.5 μm = 15 mm.

Focal plane resolution of the lens: $1 \div (2 \times 160 \text{ LP mm}^{-1}) = 3.3\ \mu\text{m}$

Amplification: $\sqrt{2123} \div 15 = 3$;

Spatial resolution: $1000 \div (3\ \times 6.5\ \ \mu\text{m} \times 2) = 25.64 \text{ LP mm}^{-1}$.

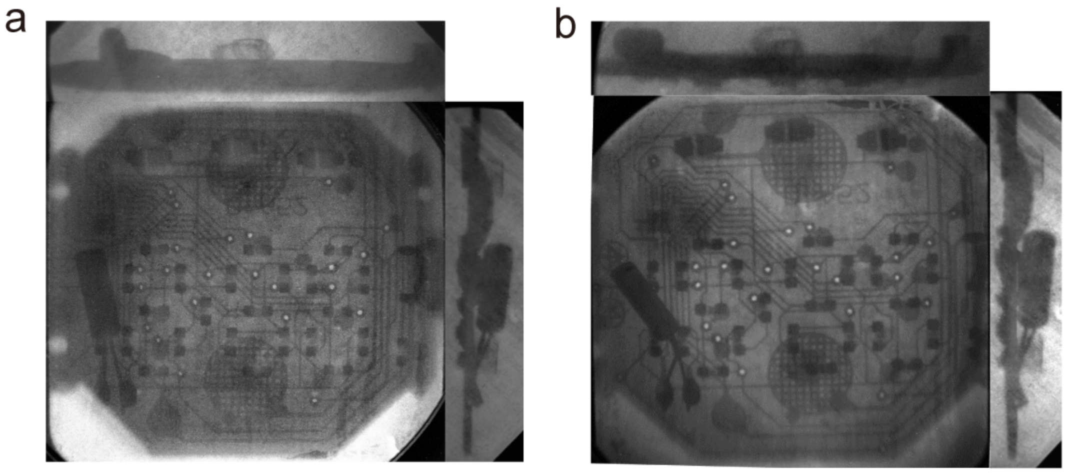

Supplementary Fig. 36 Delayed XEPL based image of the electronic watch from different directions, Lu/Tb core NPs **(a)**, Lu@Lu/Gd/Tb@Lu core@shell@shell NPs **(b)**.

Suppl. Table 1 [Na]/[RE] and activators doping ratios measured by inductively coupled plasma-optical emission spectroscopy.

Sample	[Lu]	[Gd]	[Dy]	[Na] / [RE]
Lu/Gd/Dy (2.5)	75.82	22.35	1.83	1.091
Lu/Gd/Dy (10)	76.78	21.47	1.75	1.324
Lu/Gd/Dy@Lu	88.01	11.09	0.90	1.145
Lu/Gd/Dy@Y	75.62	22.54	1.84	1.151
Y@Lu/Gd/Dy@Y	76.61	21.68	1.71	1.127

[Lu] + [Gd] + [Dy] = 100%. [RE]=[Lu] + [Gd] + [Dy] + [Y].

Supplementary references

1. Bian, W. et al. Direct Identification of Surface Defects and Their Influence on the Optical Characteristics of Upconversion Nanoparticles. *ACS Nano* 12, 3623-3628 (2018).
2. Huang, F. et al. Suppression of Cation Intermixing Highly Boosts the Performance of Core–Shell Lanthanide Upconversion Nanoparticles. *J. Am. Chem. Soc.* 145, 17621-17631 (2023).
